# Differential response of C57BL/6J mouse and DBA/2J mouse to optic nerve crush

**DOI:** 10.1186/1471-2202-10-90

**Published:** 2009-07-30

**Authors:** Justin P Templeton, Mohamed Nassr, Felix Vazquez-Chona, Natalie E Freeman-Anderson, William E Orr, Robert W Williams, Eldon E Geisert

**Affiliations:** 1 Department of Ophthalmology, University of Tennessee Health Science Center, Memphis TN, 38163, USA; 2 Department of Anatomy and Neurobiology, University of Tennessee Health Science Center, Memphis TN, 38163, USA

## Abstract

**Background:**

Retinal ganglion cell (RGC) death is the final consequence of many blinding diseases, where there is considerable variation in the time course and severity of RGC loss. Indeed, this process appears to be influenced by a wide variety of genetic and environmental factors. In this study we explored the genetic basis for differences in ganglion cell death in two inbred strains of mice.

**Results:**

We found that RGCs are more susceptible to death following optic nerve crush in C57BL/6J mice (54% survival) than in DBA/2J mice (62% survival). Using the Illumina Mouse-6 microarray, we identified 1,580 genes with significant change in expression following optic nerve crush in these two strains of mice. Our analysis of the changes occurring after optic nerve crush demonstrated that the greatest amount of change (44% of the variance) was due to the injury itself. This included changes associated with ganglion cell death, reactive gliosis, and abortive regeneration. The second pattern of gene changes (23% of the variance) was primarily related to differences in gene expressions observed between the C57BL/6J and DBA/2J mouse strains. The remaining changes in gene expression represent interactions between the effects of optic nerve crush and the genetic background of the mouse. We extracted one genetic network from this dataset that appears to be related to tissue remodeling. One of the most intriguing sets of changes included members of the crystallin family of genes, which may represent a signature of pathways modulating the susceptibility of cells to death.

**Conclusion:**

Differential responses to optic nerve crush between two widely used strains of mice were used to define molecular networks associated with ganglion cell death and reactive gliosis. These results form the basis for our continuing interest in the modifiers of retinal injury.

## Background

For many ocular diseases that result in the loss of vision, the death of retinal ganglion cells (RGCs) is the final common pathway. Glaucoma is one such ocular disease where the sporadic family history and the presence of significant risk factors in select populations suggest that the susceptibility of RGC death is a complex trait [1,2]. For example, elevation in intraocular pressure (IOP) in open angle glaucoma is strongly associated with an increased likelihood of RGC death. Lowering IOP almost always has the beneficial effect of sparing RGCs. However, some patients with normal or even low IOP develop glaucoma with associated RGC death [3,4]. The reverse is also true: selected populations of people have very high IOPs and yet do not develop glaucoma or lose RGCs [3]. The fact that some patients with low IOPs develop glaucoma while others with high IOPs do not has led to the hypothesis that critical genetic sequence variants segregating human populations influence the relative susceptibility or resistance to ganglion cell death [5]. One efficient way to measure the influence of sequence variants on complex traits is to compare different inbred strains of mice. For example, Nickells and colleagues [6] studied the differential survival of RGCs in 15 highly diverse strains of mice following optic nerve crush, finding that ganglion cells in some strains were highly susceptible whereas other strains were relatively resistant. This difference demonstrates the importance of genetic background on the complex process of ganglion cell death. Defining the genomic differences between these strains has the potential to lead to novel treatments to prevent ganglion cell loss and preserve vision.

One obvious approach to examining the molecular differences that underlie the susceptibility or resistance of ganglion cells to injury is to use microarray methods to profile the transcriptomes of inbred strains of mice. A considerable amount of published microarray data describes the retina's response to injury in different rodent strains. When one looks across all of these studies, there is a general agreement that changes in gene expression are classic responses of the central nervous system (CNS) to injury [7]. For example, genes that are associated with reactive gliosis, such as *Gfap*, are often upregulated, whereas neuronal marker genes such as *Thy1 *are often downregulated [7-13]. Some studies have gone further, focusing on the response of the inner retina to look at regional changes [14]; other studies have used laser-capture microdissection to examine expression profiles of isolated RGCs [15]. The common responses to injury can be observed in a variety of different types of insult to the eye. Earlier work in our laboratory [7] found that many of the changes resulting from mechanical injury to the eye are similar to changes in other models of retinal injury, including ischemia [16], elevated intraocular pressure [8], photocoagulation [17], and photo-oxidative stress [18]. These common responses across different rodent species with different types of insult are interesting, however since they are common to susceptible and resistant strains they are of little use in determining the underling susceptibility to insult.

In this study, we compared the response of the retina to optic nerve crush in C57BL/6J and DBA/2J mice. Both of these strains are widely used in vision research. There is an extensive catalogue of ocular phenotypes and genetic modifications for both strains [19]. Also, these two strains are the parents of the BXD recombinant inbred (RI) strain set, which has been used for more than a decade to study the genetic basis of variations in the structure of the eye, retina, and central visual system [20,21]. We have comprehensive gene expression data for the eyes of most of these BXD strains [20] and therefore can use all of these array and phenotype datasets to map sequence variants that influence entire genetic networks regulating the response of the retina to injury.

## Results and Discussion

To study molecular mechanisms underlying differential response of the retina to injury, we exploited two mouse strains, C57BL/6J and the DBA/2J. The first part of this section covers the differential effect of optic nerve crush on ganglion cell survival. For the anatomical studies the retinas were examined 30 days after the optic nerve crush. This extended period of time allowed us to have a clear picture of the long-term effects of the insult to the optic nerve. In the second part, we describe changes in the transcriptome at two time points after the crush. To define potential changes in the transcriptome that underlie the long-term effects of optic nerve crush, the microarray samples were taken at 2 and 5 days after optic nerve crush. We have supplemented the analysis by generating microarray data of cultured astrocytes from C57BL/6J and DBA/2J strains and by a meta-analysis of array data from existing public databases.

### Retinal Ganglion Cell Loss After Optic Nerve Crush

**Figure 1:**
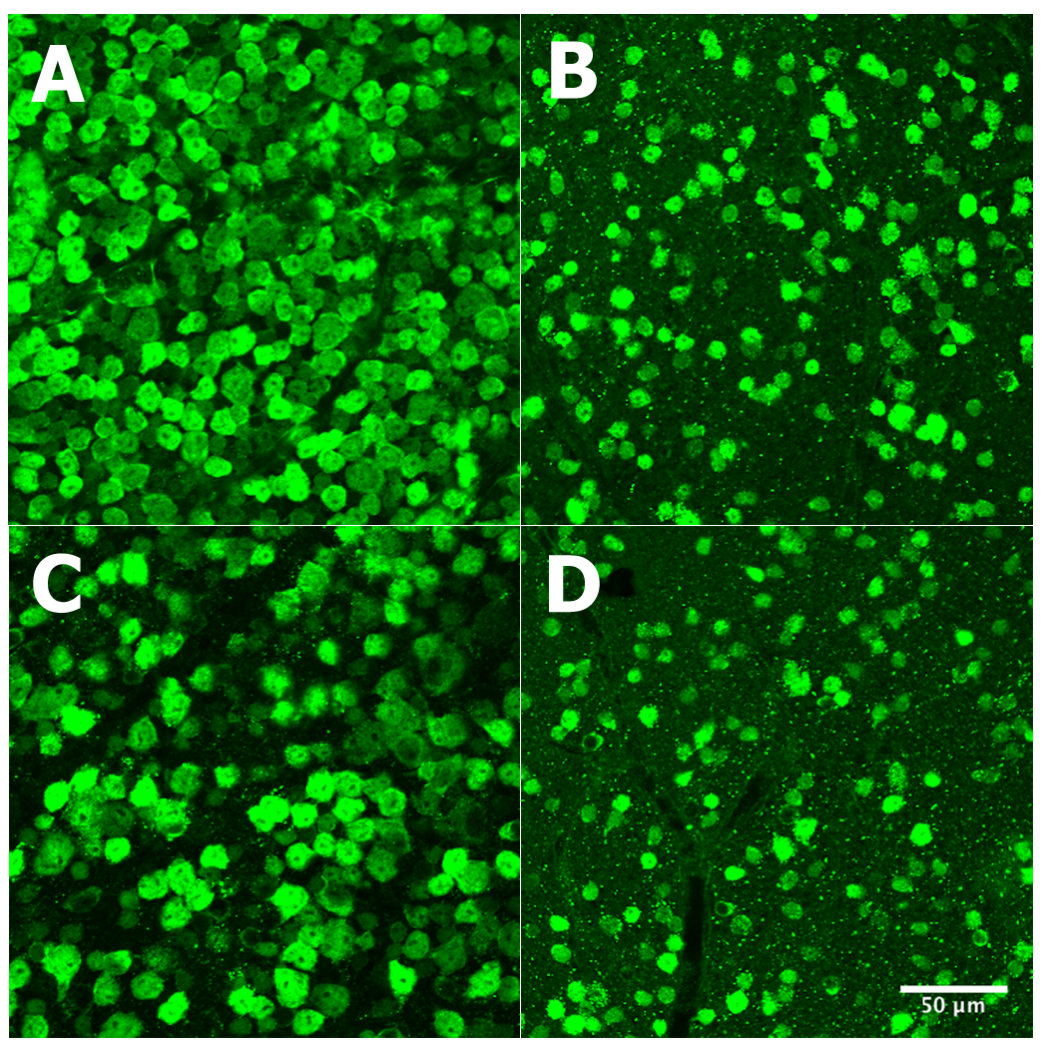
**Effects of optic nerve crush on the retinas of C57BL/6 and DBA/2J mice**. The effect of optic nerve crush on the survival of retinal ganglion cells is shown in 4 panels. C57BL/6 retinas are shown in A (control) and B (optic nerve crush); DBA/2J retinas appear in C (control) and D (optic nerve crush). Note the significant decrease in ganglion cell staining after optic nerve crush. Ganglion cells were stained with NeuN. All panels are at 40× magnification. The scale bar in panel D represents 50 μm.

To define difference in the response of the C57BL/6J and DBA/2J mice to optic nerve crush, we examined the retinas of normal mice and mice 30 days after optic nerve crush (Figure 1). The most obvious change was a dramatic decrease in the number of NeuN-stained cells in the retinas of both strains when the nerve was crushed. We counted the number of NeuN-labeled cells in the ganglion cell layers of control retinas and retinas from animals 30 days after optic nerve crush for both the C57BL/6J mouse and the DBA/2J mouse. We sampled between 14 and 18 fields from 5 retinas for each strain to define the density of NeuN positive cells per mm^2 ^(see methods section). In the control C57BL/6J mice, the average number of NeuN-positive cells (ganglion cells plus the population of displaced amacrine cells) was 5012 cells/mm^2 ^with a standard error of 317 cells/mm^2^. The control DBA/2J mice had 4295 cells/mm^2 ^with a standard error of 187 cells/mm^2^. This difference in the density of NeuN-positive cells between the two strains is significant using the student t-test (p < 0.04).

In our data, we see a significant difference in the number of NeuN-positive cells between the C57BL/6J and the DBA/2J strains. When one examines the literature for comparative numbers, Williams et al. [22] calculated the axon number to be a mean of 54,600 ganglion cells per retina for the C57BL/6J strain (pooled data from pigmented and coisoenic albino mice) and 63,400 ganglion cells per retina for the DBA/2J strain. If this is converted to cell density per mm^2 ^the numbers are 2,884 cells/mm^2 ^for the C57BL/6J strain and 3,254 cells/mm^2 ^for the DBA/2J strain. In the study by Buckingham et al. [23] they used NeuN staining and cell counting similar to that used in the present study and they arrived at very similar numbers (see Figure 2 in Buckingham et al., 2008). Extrapolating from their graph they see 4,800 cells per mm^2 ^for C57BL/6J and 4,200 cells per mm^2 ^for the DBA/2J mouse [23]. These numbers are very similar to the ones from the present study. It is hard to say why there are differences between studies. The most parsimonious explanation is that differences seen from study to study may be related to the sampling method.

**Figure 2:**
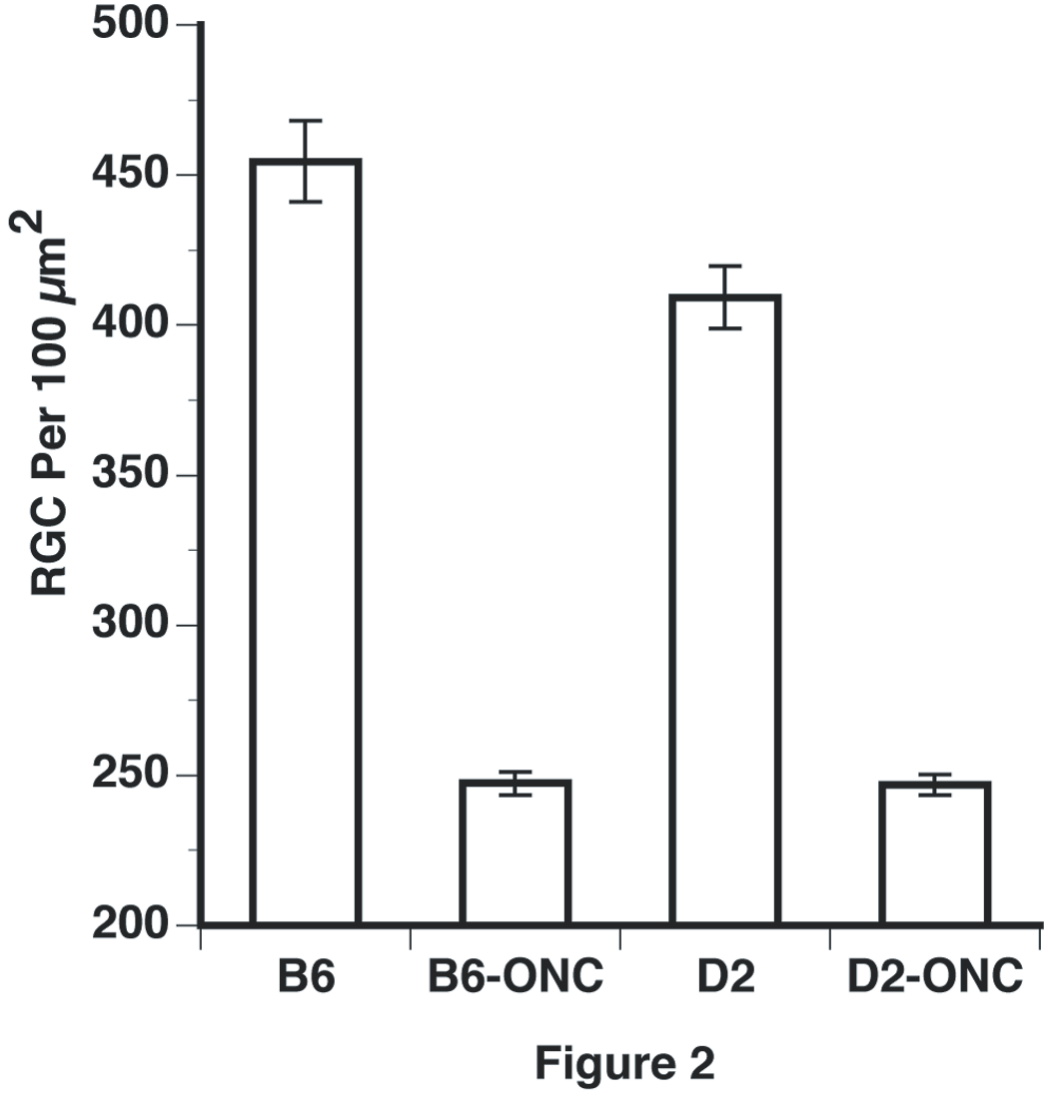
**Quantification of loss of retinal ganglion cells**. The percent survival of retinal ganglion cells following optic nerve crush (ONC) is shown for two strains of mice, C57BL/6J (B6) and DBA/2J (D2). The data points represent the percent survival for each of the retinas that received a crush of the optic nerve. The means of each group are indicated by the horizontal lines. This number was calculated by dividing the density of NeuN-positive cells optic nerve crush retinas by the average density of the control retinas of the same strain. There was a statistically significant (t-test, p < 0.01) difference in the survival of NeuN-positive cells, with 54% survival in the C57BL/6J mouse and 62% survival in the DBA/2J mouse.

After the optic nerve crush, the average density of NeuN-positive cells decreased to 2694 cells/mm^2 ^in C57BL/6J retina and to 2677 cells/mm^2 ^in DBA/2J retina. To determine if there is a difference in the response of the retinal ganglion cells to injury, we calculated the percent survival for each optic nerve crushed retina, dividing the density of retinal ganglion cells in that retina by the average density in the control retina of the same strain. The disparity in survival between the two strains is shown in Figure 2. There was a 54% survival of NeuN-positive cells in the C57BL/6J mouse, while the DBA/2J mice had a 62% survival of neurons. This difference in NeuN-labeled cell loss is significant using a student t-test (p < 0.01). Since we used NeuN to label cells in the ganglion cell layer, it is possible that displaced amacrine cells were included in our counts. The displaced amacrine cells represent a diverse population of cells [24,25]. There are strain differences in the number of amacrine cells in the mouse [26,27]. The strain differences include the number of ChaT-positive displaced amacrine cells [26]. In the C57BL/6 mouse the ChaT-positive amacrine cells are estimated to be 19% of the total population of displaced amacrine cells [28]. Thus, the neurons surviving in the ganglion cell layer may include both ganglion cells and some displaced amacrine cells. It is worth noting that Buckingham et al. [23] directly addressed this question and found that a consistent proportion of ChaT-positive displaced amacrine cells expressed detectible levels of NeuN.

When we compare our data to others, we find that Li et al. [6] did not observe a statistically significant difference in survival following optic nerve crush between the C57BL/6J mouse and the DBA/2J mouse. It is interesting to note that the DBA/2J trended higher than the C57BL/6J (see Figure 1 of Li et al.) [6]. In our study, we found a modest, but statistically significant differences between the two strains. This could be due to differences in techniques to crush the optic nerve, differences in staining, or the fact that we used control mice and Li et al. [6] used the contralateral eye as a control for optic nerve crush [6]. The small difference in survival we observe may have significant ramifications to our microarray studies and will allow us to map genetic networks using the BXD recombinant inbred mice along with the powerful bioinformatics tools in GeneNetwork [29].

### Changes in the Transcriptome After Optic Nerve Crush in the C57BL/6J and DBA/2J Strains

**Figure 3:**
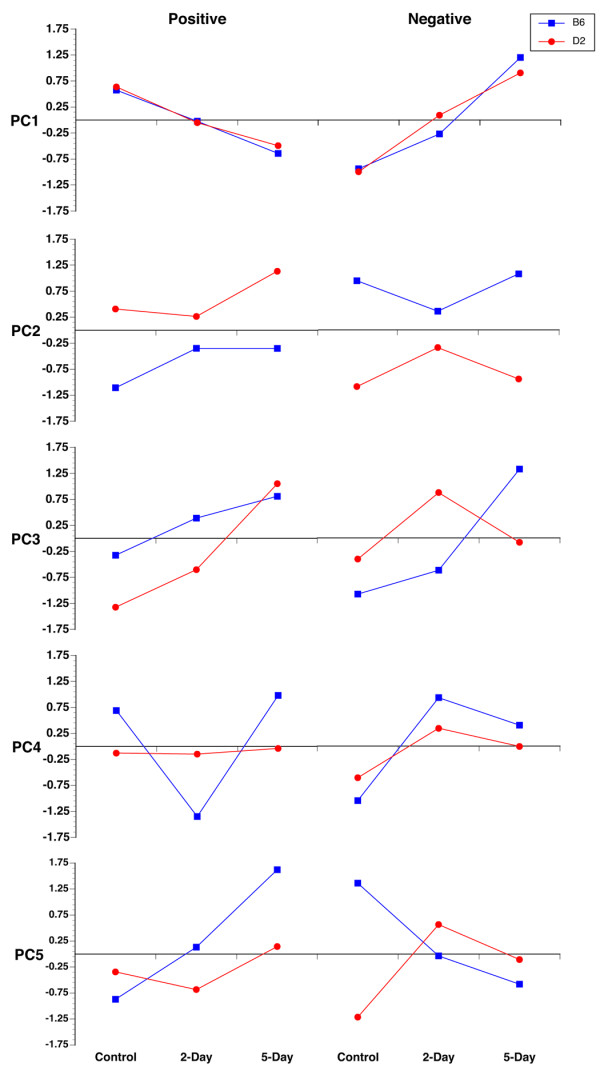
**Principal component expression patterns**. The average gene expression profiles are shown for all 10 clusters. In PC1, the genes having a common expression profile in the C57BL/6J (B6) and DBA/2J (D2) strains are clustered. PC1 positive represents genes in both strains that have decreased expression after ONC. PC1 negative has the reverse of this pattern, with genes increasing in expression. PC2 appears to represent genes that are differentially expressed in the two strains. These gene cluster patterns are almost mirror images of each other. In the remaining clusters (PC3 to PC5), very different patterns appear to represent interactions between strain differences and the effects of ONC.

Microarrays were run on both the C57BL/6J and the DBA/2J strains with RNA isolated from control mice and mice 2 and 5 days after optic nerve crush. The first step in the data analysis was to select a set of genes for analysis. For this analysis we used Significance Analysis of Microarrays (SAM, Stanford University, ). We made five biologically meaningful comparisons to define groups of genes that change in the two strains following optic nerve crush. The first comparison was to define genes that had significantly different levels of expression between the two normal retinas, control C57BL/6J retina and control DBA/2J retina (522 probes). The next four comparisons were done to select genes that were differentially expressed after optic nerve crush, comparing the control samples to the 2-day crush and 5-day crush samples of each strain for both the C57BL/6J retina and DBA/2J retina. This will define genes that are changing following optic nerve crush: C57BL/6J control versus 2 day after injury (49 probes) and versus 5 days after injury (1007 probes) and DBA/2J control versus 2 days after injury (0 probes) and 5 days after injury (52 probes). After the duplicate probes were scrubbed, a total of 1,580 probes from the Illumina array were selected with a false discovery rate less than 0.03. The next phase of the analysis was to define functional clusters of genes using a principal component analysis. Using the CLUSFAVOR v6.07 program [30], we identified clusters of genes based on their expression patterns (Figure 3). This analysis resulted in 5 eigenvectors dividing the dataset into 10 groups, with a positive and negative component for each vector (Figure 3). The 10 clusters account for all of the variability in the dataset. A number of different data-mining tools were used to extract biologically meaningful information from these clusters. The web-based software used included the National Center for Biotechnology Information web site for PubMed [31] and Entrez Gene databases [32], Chilibot for searching PubMed relationships [33], Genomatix for searching for possible transcription factors [34], Transfac 7.0 for transcription factor mining [35], UCSC Genome Bioinformatics for its genome browser [36], WebGestalt for its annotation abilities [37], Gensat [38], and GeneNetwork [29].

To complement this work, we generated a microarray dataset for astrocytes isolated from the C57BL/6J and the DBA/2J cortices. This astrocyte data set was used to generate three lists of transcripts. We used two independent approaches to create the lists. The first approach compared the average expression of all of transcripts in the 6 (3 C57BL/6J and 3 DBA/2J) astrocyte micorarrays to the average of the gene expression in the 6 normal retina samples (3 C57BL/6J and 3 DBA/2J). The first list is a group of 325 genes, which were 20-fold enriched in the astrocyte sample relative to the retina. We considered this list of genes to be an astrocyte signature. Of these 325 genes, 36 were present in the selected genes of the optic nerve crush dataset (See additional file 1: Genes with ≥ 20 fold change between astrocyte and control retina). The second list of genes, totaling 379, contained transcripts that were 20-fold enriched in the retina relative to the astrocyte sample, was classified as a non-astrocyte signature. Of these 379 genes, 44 were present in the optic nerve crush dataset (See additional file 2: Genes with ≤ -20 fold change between astrocyte and control retina). The third list of genes, totaling 934, was created to identify transcripts that were differentially expressed in cultured C57BL/6J astrocytes relative to DBA/2J astrocytes. In this list, 321 genes were more highly expressed in C57BL/6J astrocytes and 613 genes were more highly expressed in DBA/2J astrocytes. Of the 934 genes, 186 were present in the optic nerve crush dataset (See additional file 3: C57BL/6J astrocyte vs. DBA/2J astrocyte fold change). The first two of these three lists were combined and considered to be differentially expressed genes between the two types of astrocytes. These lists of genes were used in our analysis of the differential response of the C57BL/6J and DBA/2J retina to optic nerve crush.

Of the 1,580 genes in our optic nerve crush data set, 9 were selected and examined by real-time qRT-PCR. These genes were *Bcl2a1a, Chrna6, Cryaa, Crybb2, Crym, Egr1, Gfap, Sox11, and Thy1*. (See additional file 4: Oligonucleotides used for Real-Time qRT-PCR). In general, the patterns of change for the genes monitored by real-time qRT-PCR were similar to those observed in the averaged microarray datasets. The levels of *Gfap *were similar in comparison between the control data versus 2-day injury data, as well as the comparison between the 2-day versus 5-day injury data. Other genes, such as *Bcl2a1a*, *Chrna6, Cryaa, Crybb2*, *Crym*, and *Thy1*, were similar in the overall trend from control to 5-day. However, the 2-day versus 5-day fold change was not always in the same direction, although the differences were small. In other cases, there were slight differences in the fold changes, as with the *Egr1 *gene, for which the real-time qRT-PCR indicated a +0.47-fold change between the C57BL/6J 2-day versus 5-day, the microarray result was -0.8 for the same condition. *Egr1 *had a -0.35-fold change in the DBA/2J between the 2-day versus 5-day, but the microarray data yielded +1.42. Nonetheless, the agreement between the microarray data and real-time qRT-PCR data was excellent; indicating that for the genes tested, the microarray provided an appropriate measure of transcript level. Once the quality of the dataset was proven, we clustered the data into groups and began our functional analysis.

Most genes are found in the first principal component (PC1), which contains 823 genes or 44% of the total variance in the dataset. The genes in PC1 change similarly in response to optic nerve crush in both C57BL/6J and DBA/2J mice. As shown in Figure 3, the expression level for genes in PC1 is approximately equal in control C57BL/6J mice as compared to the DBA/2J mice. For PC1 positive, the genes are downregulated 2-days after optic nerve crush and even further downregulated 5-days after nerve crush. A brief scan of this list of genes (See additional file 5: Genes of principal component 1 (PC1)) reveals that many genes, such as *Thy1 *and *Chrna6*, are genes associated with ganglion cell injury and death. To provide an alternative method of determining the cell types represented in PC1 positive, we examined the labeling patterns of the GENSAT project web site [38]. GENSAT describes a series of mouse strains that label sets of cells within the mouse brain that express trans-genes under labels of gene-specific promoter constructs. Of the 407 genes in PC1 positive, 28 were found in GENSAT, with 24 genes labeling neurons in the brain and 5 (*Npc1, Pax6, Chrnb3, Htr1d*, and *Chrna6*) specifically labeling axons in the optic tract. These data indicate that PC1 positive represents genes that are involved in the generalized decrease in neuronal markers, specifically ganglion cells, in response to optic nerve crush.

PC1 negative is also composed of genes with approximately equal expression in control C57BL/6J and DBA/2J mice. However, both sets of genes are upregulated 2-days after optic nerve crush and upregulated even further at 5-days after crush (Figure 3). Genes in this group are associated not only with reactive gliosis, but with neuronal genes in an attempt at abortive regeneration (See additional file 5: Genes of principal component 1 (PC1)). Glial fibrillary acidic protein (*Gfap*), the hallmark for reactive gliosis, is found in this group. Many of the genes in this list are also markers for astrocytes. These include *Gfap*, the penultimate astrocytic cytoskeletal marker, and *Sox11 *a known astrocyte transcription factor.

To further evaluate astrocytes marker genes, we examined our data from cultured astrocytes from C57BL/6J and DBA/2J mice. In one analysis, we identified genes that were enriched in astrocytes as compared to the normal retina. Of the 36 astrocyte signature genes in our dataset of 1,580 probes, 27 are present within PC1 negative, indicating that PC1 negative contains an astrocyte signature. We also observed genes involved in abortive regeneration, among them growing axon protein 43 (*Gap43*). In examining the labeling patterns on GENSAT, 21 of the 314 genes in PC1 negative were found. Four of the PC1 negative genes (*Gfap, Stat3, Ank2*, and *Vcam1*) labeled glial cells and two of the genes (*Rax *and *Snap91*) labeled axons in the optic nerve. The changes observed in PC1 are common to CNS injury and are associated with the experimental condition of axonal injury caused by optic nerve crush. This represents the most variance in the microarray dataset and, as expected, it is due to the experimental condition. When these results are compared to other microarray studies that examined the effects of injury in the retina, there is a surprisingly similar list of genes. These are injury response elements, many of which are found in the retina after a variety of insults: ischemia [16], elevated IOP [8], photocoagulation [17], photo-oxidative stress [18], or direct retinal injury [7].

A recent publication [39] demonstrated the importance of the mTOR pathway, not only in regulating axonal regeneration in the optic nerve, but also the survival of retinal ganglion cells. A functional knockout of *Pten*, the negative regulator of mTOR, promoted axon regeneration and the survival of RGCs after optic nerve injury [39]. In our dataset, we observed various changes in 25 genes within the PTEN/mTOR pathways. Furthermore, PTEN itself is significantly upregulated and is in PC1 negative. These findings clearly show that some of the molecular changes associated with the abortive regenerative response of the PTEN/mTOR pathways are associated with the changes we observed in our study. The presence of 14 of these genes in PC1 positive indicates that they have a generalized function in the abortive response of RGCs. Future studies will focus on the importance of this pathway following injury to the optic nerve.

The next principal component (PC2) appears to group genes having different levels of expression in the two strains of mice. PC2 contains 496 genes, which represents 23% of the variance in the dataset (See additional file 6: Genes of principal component 2 (PC2)). The most obvious difference is the level of gene expression between the C57BL/6J control group and the DBA/2J control group (Figure 3). In PC2 positive (223 probes), the expression level in C57BL/6J mice is low, while the DBA/2J mice have high levels of expression. Furthermore, the pattern of expression after optic nerve crush in the DBA/2J mouse is virtually a mirror image of the C57BL/6J, with expression decreasing 2-days after crush in the DBA/2J mice and increasing at 2-days after crush in the C57BL/6J mice. The PC2 negative (270 probes) is almost the reverse of the PC2 positive component, with the expression level in C57BL/6J mice being high and the expression level in DBA/2J mice being low. To investigate further, we rank-ordered the fold change between the C57BL/6J and DBA/2J control retinas. Among the genes in PC2 in this ranked list, 407 (82%) of the 496 probes were in the top 5% of the most variable genes in the dataset. The most parsimonious explanation is that the genes in PC2 are differentially expressed in the C57BL/J and DBA/2J retinas and are not differentially affected by optic nerve crush.

To test the hypothesis that PC2 represents genes that differ between the two strains, we examined the Hamilton Eye Institute Mouse Eye Database (HEIMED) on GeneNetwork [20] to determine whether any of the genes in PC2 had a significant quantitative trait locus (QTL) or were part of a gene network within the eye. Most of the genes had strong cis-QTLs and were not part of an overall genetic network. The large cis-QTL found in GeneNetwork also points to the fact that these are genes with differences in expression in C57BL/6J and DBA/2J mice, the parental strains of the BXD RI strain set. The gene network analysis and expression patterns led us to the same conclusion.

This being the case, one would predict that these differences are present in different tissues from these two strains. Thus, we examined the microarray databases generated from C57BL/6J and DBA/2J cultured astrocytes. A surprising number of genes are differentially expressed between the C57BL/6J and DBA/2J astrocytes and are also present in PC2. Of the 184 differentially expressed astrocyte genes found among the 1,580 genes of the Optic Nerve Crush dataset, 162 were in PC2. This indicates that the differential expression between the two strains is also in astrocytes as well as the retina. Furthermore, genes that are more highly expressed in DBA/2J astrocytes are highly expressed in the DBA/2J retina, while genes that are expressed at higher levels in C57BL/6J astrocytes are highly expressed in the C57BL/6J retina.

If PC2 represents genes that are differentially expressed, one would predict that these genes would be expressed in a variety of different cell types which is in fact, the case. The genes in PC2 are found in all cell types within the CNS. A total of 30 genes were found in the GENSAT database. In PC2 positive, 3 genes (*Slc7a14, Cacng5*, and *Susp3*) were expressed in optic nerve axons and neurons. In PC2 negative, one gene (*Casp9*) was observed labeling axons in the optic nerve. In addition, neurons were labeled in the brain by 9 additional genes, including *Mtap1b, Lypd1, Cdon, D430039no5Rik, Dap3, Dcnq2, Rgs16, Tac2*, and *Tph2*. Both PC2 negative and positive contained glial genes; 2 (*Sf1 *and *H2-D1*) were in PC2 positive and 4 (*Dusp16, Fcer1g, Prom1*, and *Hdc*) were in PC2 negative. Thus, PC2 appears to represent genes that are differentially expressed in the C57BL/6J and DBA/2J retinas. These genes do not appear to be related to any specific cell type or function.

The most intriguing functional components are within PC3, PC4 and PC5 (representing 33% of the variance in the dataset) (See additional file 7: Genes of principal component 3 through 5 (PC3-PC5)). These components represent interactions between the effects of optic nerve crush injury and the genetic background of the two mouse strains. If there is a signature for susceptibility or resistance to ganglion cell death, or if there is a signature of reactive gliosis, then it lies within these components. Accordingly, we used all of the bioinformatics tools at our disposal to identify a meaningful association between the genes within each component. First, to determine if there was a molecular signature of a specific cell type within any of the PCs, we searched cell-type-specific profiles in Cahoy et al. [40], who provided databases of genes enriched in astrocytes, neurons, and oligodendrocytes. We were not able to identify a signature for a specific cell type in our microarray datasets. We also used online databases NEI Bank [41], WebGestalt [37], GeneNetwork [29], and Gene Ontology [42] but were unable to identify clear functional associations of the genes within most of the PC3 to PC5 clusters. In two clusters, PC3 negative and PC5 negative, we were able to impart functional associations between the genes.

Our laboratory is one of the first to use the bioinformatics tools on GeneNetwork to explore the functional relationships between genes clustered in a microarray experiment [43,44]. The basic hypothesis is that common regulatory elements modulate genes to create similar changes, thereby driving them into the same PC. A similar approach was taken with the data in the current study. We loaded all of the data from the individual PCs into GeneNetwork and examined it to determine whether genomic loci could be identified that modulated the expression of a significant number of genes within a given PC. We used two databases, the BXD eye database (Eye M430v2, Sep08 RMA Database) and the BXD striatum database (HQF BXD Striatum ILM6.1, Nov07 RankInv Database), which was run with the same Illumina chip used in the present study.

**Figure 4:**
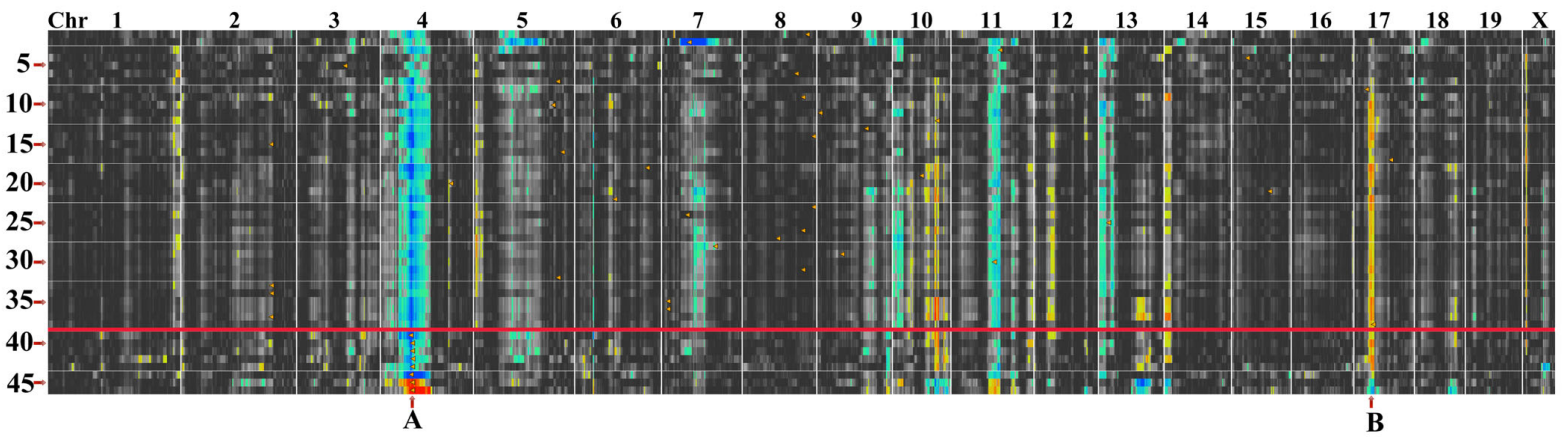
**Quantitative trait locus cluster map of PC3 negative**. The QTL cluster map for genes in PC3 negative shows potential regulatory genomic loci. Horizontal heat maps represent individual genes. There are 38 genes (those above the red line) in the PC3 negative network in this heat map; the numbers to the left denote the individual genes. The genes are represented in a genome-wide scan with the chromosomal locations at the top of the heat map ranging from Chr 1 on the left to Chr X on the right. The yellow, red, and blue bands denote the locations of genomic loci that modulate all of the genes in the network. Note that some bands are more prominent than others. The two most prominent bands are in Chr 4, 50-105 Mb (A) and Chr 17, 27-40 Mb (B). The network analysis can be taken one step further by identifying candidate genes within the loci, using advanced search tools on GeneNetwork. For the regulatory locus on Chr 4 (A) the genes are *Ltb4dh, Pole3, Rgs3, Tnc, Ugcg, and Fkbp15*. A candidate gene for Chr 17(B), *Bat1*, is from our PC3 negative group. Note that these candidate genes have a QTL structure (banding pattern) similar to that of genes within PC3 negative.

Only PC3 negative was found to have a series of genomic loci modulating the expression of the genes and this was observed only in the striatum database. Of the 57 genes in PC3 negative, 43 were found to have common regulatory genomic loci (Figure 4). The genes and loci shown in Figure 4 may represent a genetic network that is activated by optic nerve crush. The bands seen in the QTL-heat map represent the likelihood ratio statistic (LRS) and provide a measure of the linkage between variation in the phenotype and genetic differences at a particular genetic locus [45]. As the color progresses from yellow to red or from green to blue, the LRS score increases. Some genes in PC3 negative (*Anapc5, Atp6v0d1, Aup1, Bat1a, Cdk10, Csda, Eef2, Map1lc3a, Nubp1, Prmt7, Ranbp3, Snrpa, Snrpn, Trafd1, Trpc4ap, Tspan3*, and *Wrnip1*) have a LRS to Chr 4 (Figure 4). Individually, the remaining genes do not have significant LRS scores; collectively, however, they form a pattern that reveals a modulatory genomic network (See additional file 8: Network map of PC3 negative along with candidate genes). This banding pattern (Figure 4) is a signature of loci modulating most of the genes in PC3 negative.

With GeneNetwork, we are able to examine the trans-bands in the PC3 negative QTL map to define candidate genes that may be upstream modulators of the network. We selected two bands for this analysis, Chr. 4 and Chr. 17. Using the advance search program on GeneNetwork, the loci were mined to identify genes within them that have significant QTLs (Cis-QTLs). Six candidate genes (*Ltb4dh, Pole3, Rgs3, Tnc, Ugcg*, and *Fkbp15*) with Cis-QTLs in Chr 4 50-105 MB were found. One candidate gene, *Bat1a*, (See additional file 9: Candidate genes for PC3 negative QTL) was identified for the band on Chr. 17 27-40 MB. On examination, *Bat1a *was found to be one of the genes within the PC3 negative gene cluster (Figure 4). Thus, it appears that PC3 negative forms a functional gene network with a yet unknown function. To find the function of the PC3 negative gene network, we ran the list of genes on WebGestalt and Gene Ontology. WebGestalt showed that 20 genes were involved in cellular metabolism, with 10 of the 20 (*Bat1a, Snrpa, Csda, Snrpb, Krr1, Wrnip1, Snrpn, Prmt7, Mtdh*, and *Atp6v0d1*) involved in nucleic acid metabolism and 9 of the 20 (*Anapc5, Cdk10, Uble1a, Eef2, Ptp4a2, Fbxl12, Map1lc3a, Prmt7*, and *Eif5a*) involved in cellular macromolecule metabolism. The gene ontology for the group found that 4 genes (*Map1lc3a, Uble1a, Anapc5*, and *Fbxl12*) had a biological process in the ubiquitin cycle and 20 other genes (*Cdk10, Clns1a, Cope, Coq5, Csda, Ergic3, Etfb, Gbas, Krr1, Map1lc3a, Mapk8ip3, Mdm1, Mtdh, Prmt7, Snrpb, Snrpn, Sv2a, Trpc4ap, Uble1a*, and *Wrnip*) had a cellular function relating to an intracellular membrane-bound organelle. Based on the biological function, the genes clustered in PC3 negative appear to be related to cellular remodeling.

**Figure 5:**
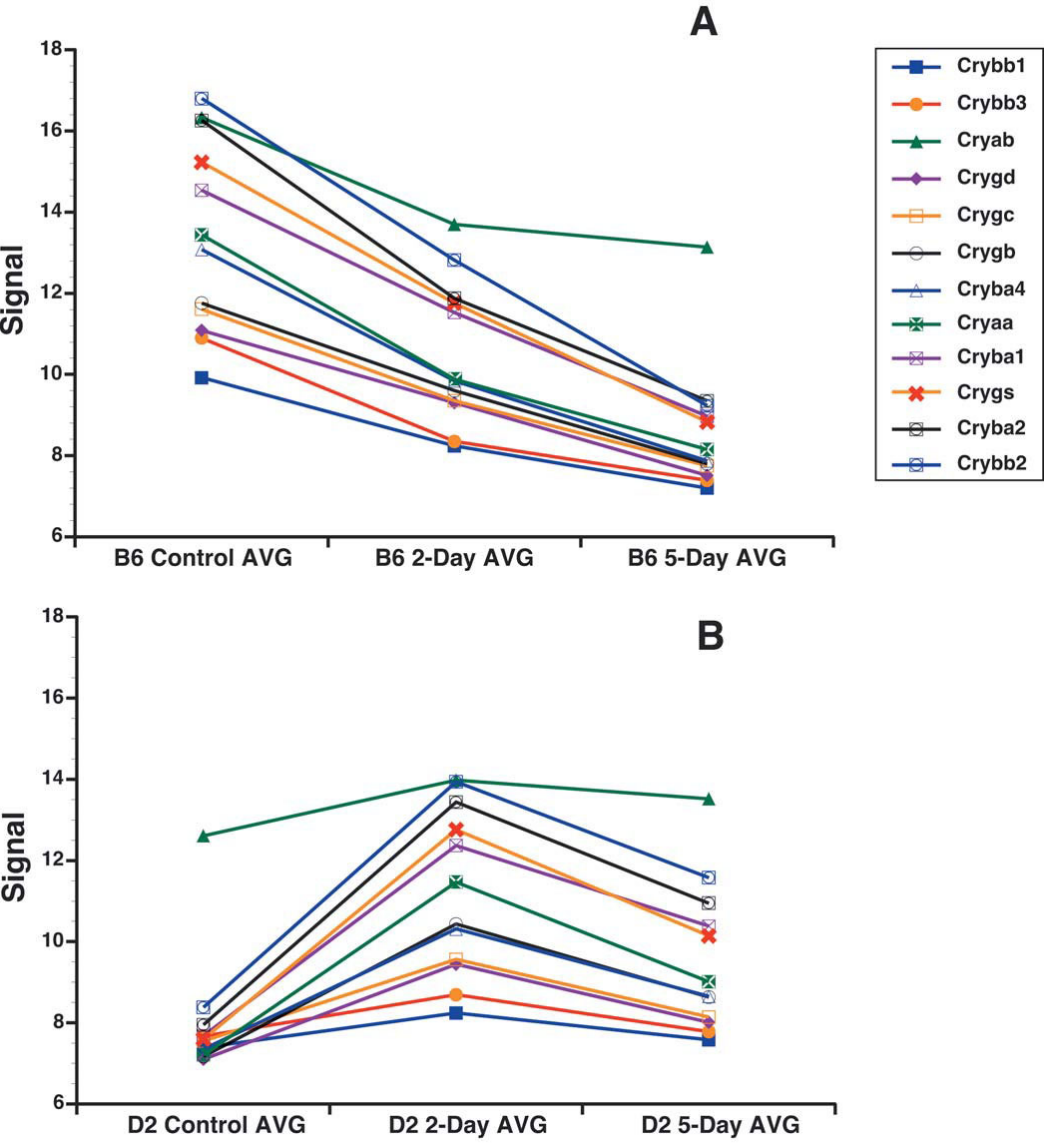
**Graph of crystallin genes comparison between the strains and experimental conditions**. The relative fold changes in selected crystallin mRNA for the C57BL/6J (B6) strain (A) and DBA/2J (D2) strain (B) are illustrated. To the far right is a legend identifying the specific crystallin family members. The members of the crystallin family with this pattern of expression include and significant changes are Cryaa, Cryab, Cryba1, Cryba2, Cryba4, Crybb1, Crybb2, Crybb3, Crygb, Crygc, Crygd, and Crygs. This was not the case with all the crystallin genes. Notice that in general the crystallins are more highly expressed in the normal C57BL/6J retina than in the normal DBA/2J retina. After injury, the expression level of the crystallins decrease in the C57BL/6J while in the DBA/2J retina the levels increase. Signal is expressed as a 2 (z-score of log2 [intensity]) + 8.

**Figure 6:**
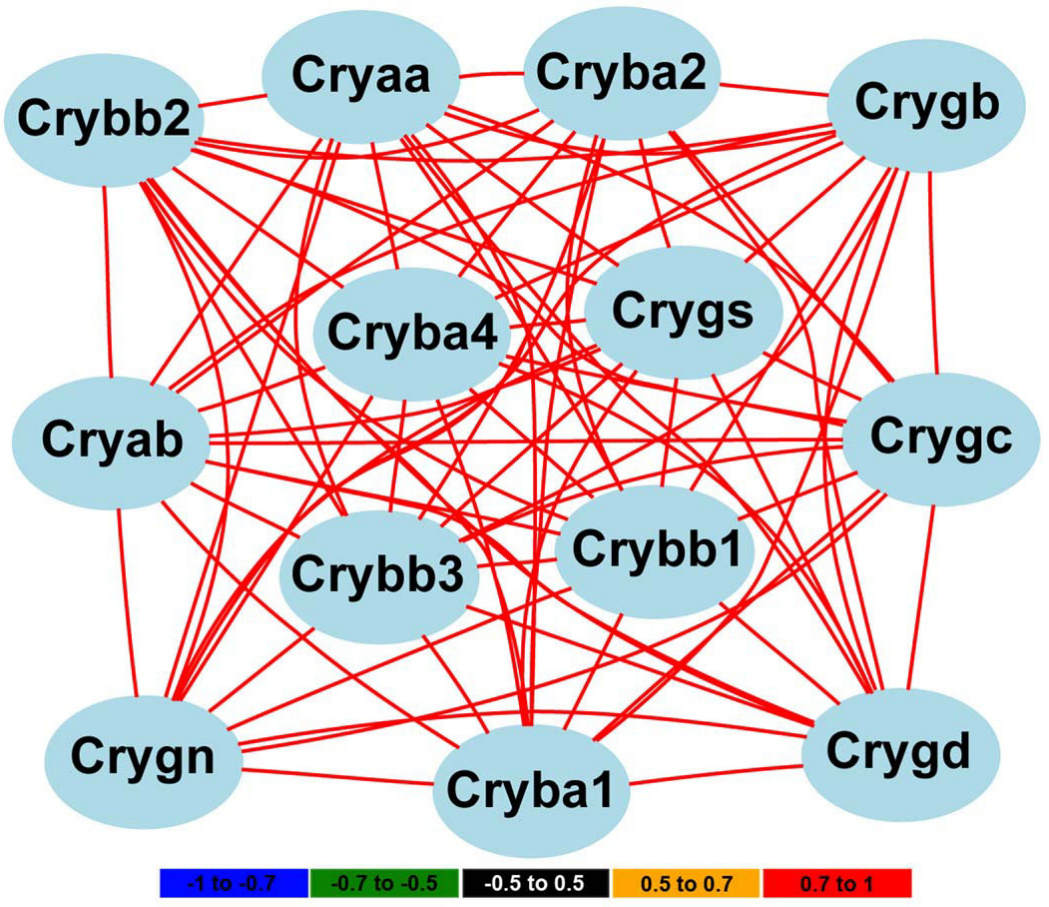
**Correlation of crystallin genes expression in the normal retina of the BXD RI strain set**. The expression patterns of many of the crystallin family members across the 49 BXD RI strains in the HEI Retina Database are highly correlated. The color of the lines connecting gene symbols indicate the degree of expression correlations (see scale at bottom of the figure). For these genes the correlation between the group is high (r > 0.7).

One intriguing finding was the presence of crystallin genes in PC5 negative. Crystallin family members in C57BL/6J mice respond to optic nerve crush differently than do those in DBA/2J mice (Figure 5). This observation led us to reanalyze the entire crystallin family. We found that many of the crystallin family members have a similar pattern of expression, being downregulated in the C57BL/6J retina and upregulated in the DBA/2J retina at 2-days after optic nerve crush. The members of the crystallin family with this pattern of expression include *Cryaa, Cryab, Cryba1, Cryba2, Cryba4, Crybb1, Crybb2, Crybb3, Crygb, Crygc, Crygd*, and *Crygs *(Figure 5). Thus, it appears that a selective group of crystallins is differentially affected by optic nerve crush in the two strains of mice. If we examine the HEI retina database for the genes that are differentially modulated by optic nerve crush (see Figure 5), it is very clear that the expression of this group of crystallins is highly correlated across the strains in the BXD RI strain set (Figure 6). This is a strong indicator that this subset of crystallin genes form a genetic network within the normal retina and the differences we observed in the parental strains (C57BL/6J and DBA/2J) are the basis for the network observed in the BXD retinas. One could argue that this is due to lens contamination or an oddity of the retina; however, this is not the case. If we examine the BXD hippocampus dataset in GeneNetwork, then we can see a similar highly correlated network (Table 1). This would exclude the possibility of contamination by the lens, for it is not present within the brain. Furthermore, the crystallin network does not appear to be specific to the BXD RI strain set. A similar crystallin network is also observed in the LXS RI hippocampus (Table 1). Thus, the crystallin network is found in the hippocampus of two different RI strain sets, the BXD and the LXS. The crystallin network is not an artifact of the microarray platform being used. In the BXD retina dataset we used the Illumina Sentrix Mouse 6 version 2.0 array, in the BXD hippocampus dataset we used the Affymetrix Mouse Expression 430 v2.0 array, and in the LXS hippocampus we used the Illumina Sentrix Mouse 6 version 1.0 array. This indicates that crystallin network is found using very different microarray platforms and is not due to an inherent complication that is platform specific. Finally, this crystallin network is not generalized to all CNS structures. If we examine the BXD cerebellum dataset or the BXD striatum dataset in GeneNetwork, there is no correlation between these crystallin family members.

**Table 1:** Crystallin gene correlation across strains and tissues

**Gene Symbol**	**Gene Name**	**Tissue and Database**		
		**BXD: Retina**	**BXD: Hippocampus**	**LXS: Hippocampus**

Cryaa	crystallin, alpha A	0.981	0.940	0.868

Cryba1	crystallin, beta A1	0.998	0.973	0.974

Cryba2	crystallin, beta A2	0.999	0.627	0.988

Cryba4	crystallin, beta A4	0.988	0.844	0.963

Crybb1	crystallin, beta B1	0.953	-	0.579

Crybb2	crystallin, beta B2	0.997	0.899	0.976

Crybb3	crystallin, beta B3	0.952	0.682	0.927

Crygb	crystallin, gamma B	0.882	0.913	0.988

Crygc	crystallin, gamma C	0.888	0.948	0.960

Crygd	crystallin, gamma D	0.858	0.828	0.980

Crygs	crystallin, gamma S	1.000	1.000	1.000

The table contains the crystallin genes, which showed the expression pattern due to injury seen in Figure 5. The values represent the correlation values in our HEI Retina Database for the BXD RI strain set. The correlation values are also shown for Hippocampus tissues in the BXD RI strain set as well as the LXS mouse cross. This demonstrates that the crystallin gene correlation is not limited to the retina or the BXD. Correlation values are related to the probe or probe set for Crygs.

Is this difference in crystallin response unique to these mice or is it found in other species? Examining the literature, we found that the response of crystallins in the retina varies from strain to strain in both mouse and rat. For example, in Wistar [46] and Brown Norway rats [8], crystallin expression goes down after elevated IOP. In Sprague-Dawley rats, crystallins are upregulated after either a retinal tear [7] or after light damage [47]. A similar story is found for the mouse. In the naturally occurring glaucoma of the DBA/2J mouse, Steele et al. [48] found that crystallins are downregulated. In the C57BL/6 mouse, crystallins are upregulated after laser burns in the retina [17]. Taken together, these findings clearly demonstrate different responses of the crystallin family depending on the strain of rodent as well as different types of insult.

Initially one thinks of crystallins as lens proteins; however, it is becoming increasingly clear that members of this family have biological roles outside the lens [47,49-53]. Several studies of the retina have demonstrated a group of crystallins that are altered by light-induced injury [47], elevated IOP [8,54], or retinal detachment [16]. Changes in crystallin expression is also found after injury in other regions of the CNS, including the spinal cord [55] and cerebral cortex [56]. Selected crystallins also have a function in the response to injury in non-neuronal tissues such as heart muscle [57,58] and kidney [59]. Thus, the crystallins can no longer be thought of as merely lens proteins, but must be considered as having a larger biological role.

What function do crystallins have in the retina and other tissues? The α-crystallins possess chaperone activity and share properties with heat-shock proteins [50,60]. The β-crystallins and γ-crystallins also respond to stress in tissues other than the lens and have an AP-1 binding site in their promoter regions [61]. Most functional studies of crystallins point to an intrinsic action of the proteins affecting intracellular molecular processes. Specifically, α-crystallins appear to have anti-apoptotic activity [62-66]. Recently, Fischer et al. [67] suggested that purified crystallins added to the vitreous chamber or cultured explants of retina promote axonal growth and induce reactive gliosis. In the present study, we observed differential regulation of crystallin family members following optic nerve crush in C57BL/6 and DBA/2J mice. It is tempting to speculate that this differential expression of crystallins may account, in part, for the differential survival of retinal ganglion cells after optic nerve crush. This hypothesis is supported in part by recent studies demonstrating that crystallin family members protect retinal ganglion cells from the effects of optic nerve crush [68].

Inbred mouse strains are powerful tools in defining the genetic basis for selective susceptibility to neuronal death. By examining differences in the susceptibility of individual mouse strains, it is possible to identify the contribution of genetic background to differential neuronal death. The genetic backgrounds of inbred strains influence the response of the retina and CNS to disease and injury. A prime example of this approach is a paper from the Nickells group [6]. The authors examined neuronal survival following optic nerve crush in 15 strains of mice, finding that a substantial difference in neuronal survival depended on the mouse strain studied. The most resistant strain was the DBA/2J mouse; the most susceptible was the BALB/cByJ mouse. The strain hierarchy of the effect of nerve crush does not translate to other types of CNS neurons. In the spinal cord, neurons in the BALB/c are highly susceptible to contusion injury [69], whereas neurons of the substantia nigra in the BALB/c strain are relatively resistant to 1-methyl-4-phenyl-1,2,3,6-tetrahydropyridine (MPTP), as well as experimental autoimmune encephalitis (EAE) [49,70,71]. In the C57BL/6J mouse strain, retinal ganglion cells are comparatively resistant to optic nerve crush but are susceptible to MPTP exposure and EAE. Interestingly, the C57BL/6J and BALB/c strains are approximately equal in their susceptibility to spinal cord injury [69]. Schauwecker and Steward [72] determined that the C57BL/6J and BALB/c strains are invulnerable to glutamate-induced excitotoxic cell death in the hippocampus. The complex effects of genetic background on the vulnerability of neurons to injury are revealed by each strain's response, which is also dependent on the specific type of insult or the CNS region that is injured. We found small but statistically significant differences between two closely related strains, the DBA/2J strain and the C57BL/6J strain [73].

## Conclusion

In conclusion, this study defines the differential responses of the DBA/2J mouse and the C57BL/6J mouse to optic nerve crush. These include genetic networks that are associated with ganglion cell death and reactive gliosis. These findings from different inbred strains support the notion that the differential susceptibility of neurons to injury is a complex genetic trait modulated by several distinct genomic loci. In fact, several genomic loci have been mapped in the mouse that modulate the response of neurons to injury [74,75]. The first QTL mapped for neuronal injury in the mouse, on distal chromosome 1 [76], implies susceptibility to MPTP-induced death of substantia nigra neurons. Recently, a locus that modulates the response of retinal ganglion cells to axon damage (optic nerve crush) was mapped to chromosome 5 [74]. In addition, a QTL in the hippocampus that modulates excitotoxic susceptibility to cell death was mapped to distal chromosome 18. Thus, at least three loci are known that can modulate the susceptibility of neurons to injury. Each locus was mapped using different types of neurons (in different region of the CNS) and a different type of insult (ranging from crushed axons to chemical insult). Nonetheless, we can see that different regions of the genome affect neuronal survival. There is every reason to believe that this complex trait will include even more loci to produce specific susceptibility phenotypes among different strains of mice.

## Methods

Mice were used in three separate sets of experiments. We used 11 C57BL/6J mice and 10 DBA/2J mice between 60 and 90 days of age for anatomical studies to determine the percentage of neurons surviving after optic nerve crush. For the microarray studies 9 C57BL/6J mice and 9 DBA/2J mice from 60 to 90 days of age were used with 3 mice (two pooled retina samples) for each dataset. The astrocytes were cultured from 3 C57BL/6J and 3 DBA/2J mice at postnatal day 3 (P3) to produce six independent samples of cerebral astrocytes, following procedures detailed by Geisert and Stewart [77].

### Optic nerve crush

Mice were deeply anesthetized with a mixture of 13 mg/kg of Rompum and 87 mg/kg of Ketalar. A small incision was made in the lateral aspect of the conjunctiva. With a pair of small forceps the edge of the conjunctiva next to the globe was retracted slightly and rotated laterally, allowing visualization of the posterior aspect of the globe where the optic nerve could be observed. Viewed under a binocular operating microscope, the surrounding connective tissue and muscle was gently separated from the nerve. The exposed optic nerve was grasped for 10 sec with a pair of Dumont cross-clamp #7 forceps (Roboz, cat. #RS = 5027, Gaithersburg, MD). This instrument was chosen because its spring action applied a moderate yet constant and consistent force to the optic nerve. The forceps were then removed and the eye was allowed to rotate back into place. The Animal Use Committee at the University of Tennessee Health Science Center approved all procedures for the surgery and handling of mice.

### Immunohistochemical Analysis of Retinal Ganglion Cell Loss

To evaluate the number of retinal ganglion cells following optic nerve crush, we used 5 C57BL/6J mice and 5 DBA/2J mice (8 retinas per strain) as controls. We performed crush injury to the optic nerve of 6 C57BL/6J mice and 5 DBA/2J mice (12 and 8 retinas, respectively.) We crushed both nerves on the experimental animals. Several studies [78,79] have shown that crushing the optic nerve unilaterally has measurable effects on the contralateral retina. For this reason we have chosen to use one group of mice for optic nerve crush and a second naive set of mice as controls. The mice receiving optic nerve crush were allowed to survive for 30 days, after which they were deeply anesthetized with a mixture of 13 mg/kg of Rompum and 87 mg/kg of Ketalar, then perfused through the heart with saline followed by 4% paraformaldehyde in 0.1 M phosphate buffer (pH 7.3). We post-fixed the eyes for 24 hours. The next day the retinas were dissected free from the globe and rinsed in phosphate buffered saline. The intact retinal cups were placed in citrate buffer (10 mM sodium citrate, 0.05% Tween 20, pH 6.0) at 90-100°C for 20 min. The retinas were extensively rinsed in PBS, and then placed in 2% bovine serum albumin (BSA) for 30 min. The retinas were stained with primary antibodies directed against NeuN (1:250 monoclonal mouse anti-neuronal nuclei, Millipore, Billerica MA). The retinas remained in the primary antibodies for two days at 4°C. After three extended rinses in PBS, the retinas were transferred to secondary antibodies in 2% BSA for two days. Alexa Fluor 555 donkey anti-mouse IgG (Invitrogen Molecular Probes, Eugene OR) was used to stain the NeuN antibody. After rinsing the retinas, four small cuts were made in each one to assist in the retinal flat mounts. Each retina was flooded with Fluoromount-G and covered with a coverslip.

Low-power (4×) photomicrographs of the slides were taken using the Nikon Eclipse TE2000-E confocal microscope along with 1 mm scale bar. The area of each retina was calculated using the scale bar and NIH Image-J software. To determine the density of ganglion cells in a given retina, we placed a sampling grid over the 4× image of the retina in an orientation that maximized fields across all regions of the retina. Then we photographed the retina at each of the intersect points in the grid. This resulted in a minimum of 14 or a maximum of 18 sampling fields per retina. The NeuN-positive cells in each field were counted to define the number of ganglion cells per field. We used the mean number of ganglion cells per field to define the percent of change in retinal ganglion cells following optic nerve crush.

### Illumina Microarray Methods

The Illumina Sentrix Mouse-6 BeadChip V1.0 interrogated approximately 46,000 sequences from the mouse transcriptome. We used three samples from each strain and condition. For each sample, the RNA was pooled from two retinas. The tissue was homogenized and extracted according to the RNA-Stat-60 protocol as described by the manufacturer (Tel-Test, Friendswood, TX). The quality and purity of RNA was assessed using an Agilent Bioanalyzer 2100 system. The RNA from each sample was processed with the Illumina TotalPrep RNA Amplification Kit (Ambion, Austin, TX) to produce labeled cRNA. The cRNA for each sample was then hybridized to an Illumina Sentrix^® ^Mouse-6-V1.0 BeadChip (Illumina, San Diego, CA). A total of three Beadchips were used for the experiment. The Sentrix Mouse-6-V1 BeadChip contains ~48,000 probe sets directed against approximately 44,000 transcripts. Raw image data was subjected to quality control analysis using Illumina BeadScan software. MIAME standards were used for all microarray data. The data discussed in this publication have been deposited in NCBI's Gene Expression Omnibus [80] and are accessible through GEO Series accession number GSE17117 .

Once data was collected from all experimental conditions, we normalized the data using the formula 2 (z-score of log2 [intensity]) + 8 as previously described [7,81]. This procedure sets the mean expression level across a single microarray to 8 units on an exponential scale similar to that produced by real-time qRT-PCR. For the microarray analysis, we compared the changes in the transcriptome of C57BL/6J mice to that of DBA/2J mice before and after optic nerve crush. The mice, at 60-90 days of age, could be considered adults with fully developed retinas. At this age range, DBA/2J mice had not yet developed symptoms associated with pigmentary dispersion glaucoma. For each mouse strain, three independent samples were run for each experimental condition: control retina RNA and RNA isolated from retinas 2-days and 5-days after optic nerve crush. The microarray data formed a 2 by 3 experimental comparison; with optic nerve crush representing one dimension of the comparison and the two mouse strains representing the other dimension.

To test the quality of the data generated, we selected the top 100 transcripts with the least change between control and experimental C57BL/6J mice 2-days after optic nerve crush. In essence, these transcripts function as 100 housekeeping genes. When these transcripts are examined across all six experimental conditions, identical patterns of expression are observed, with these transcripts expressed at equivalent levels in C57BL/6J and DBA/2J mice. The 100 probes were selected from the C57BL/6J control set as compared to the 2-day injury dataset (r^2 ^= 0.99). Across all of the parametric comparisons within the group of six conditions, the worst fit for these 100 genes was between the DBA/2J control and experimental datasets and the C57BL/6J 5-days after optic nerve crush dataset. In this case, the bivariate correlation was still high (r^2 ^= 0.68). The quality and stability of this dataset is extremely good. There was no change between animals in probes recognizing low levels of expression (a value of 7 on our scale, which represents 1 log_2_, 2-fold, below the mean expression level) or probes having high levels of expression (a value of 16 on our scale, which represents 8 log_2_, 128-fold, above the mean expression level).

We used Significance Analysis of Microarrays (Significance Analysis of microarrays [SAM] v3.0 Stanford University, ) to calculate a list of transcripts we considered to be differentially expressed across our datasaet. We examined differences between the control samples and between the controls and either 2 days after injury or 5 days after injury for each strain. All of the transcripts had a false discovery rate below 0.03. The duplicates were scrubbed resulting in a final 1580 genes. The 1,580 SAM significant transcripts were entered into CLUSFAVOR 6.0 (Departments of Medicine, Molecular and Human Genetics, and Scott Department of Urology, Baylor College of Medicine, Houston, TX) for pattern analysis.

### Microarray Confirmation Through Real-Time qRT-PCR

Selected genes from the microarray datasets, including *Bcl2a1a, Chrna6, Cryaa, Cryba2, Crybb2, Crym, Egr1, Gfap, Lpin1, Rho, Sag, Sox11, and Thy1 *as well as *Actb *as a control, were validated using real-time qRT-PCR, which was done on the Roche LightCycler 480 system (F. Hoffmann-La Roche, Switzerland). To normalize the data, *Actb *was run as a housekeeping gene. Each gene was run under 5 concentrations in duplicate along with negative control, neg-RT, as well as water for controls. Assays were designed on the Roche web site [82]. The primers were synthesized by Integrated DNA Technologies (See additional file 4: Oligonucleotides used for Real-Time qRT-PCR).

## Authors' contributions

JPT performed the real-time qRT-PCR, optic nerve crush on samples used for staining as well as contributed to the writing of all sections of the manuscript. MN continued FV's work and performed the optic nerve crush, RNA isolation, running and analyzing the microarray data, as well as contributing to the writing of the manuscript. FV participated in the beginning design of the study and helped refine the desired procedure of optic nerve crush for this manuscript. NEA ran the microarray data on the retina database as well as bioinformatics on the crystallin network. WEO performed the statistical calculations and analysis, on the microarray data. RW contributed his knowledge of GeneNetwork and the analysis of the results as well as participated in writing the discussion section of the manuscript. EEG conceived the study, and participated in its design, coordination and drafting the manuscript. All authors read and approved the final manuscript.

## Supplementary Material

Additional file 1 Genes with ≥ 20 fold change between astrocyte and control retina Click here for file

Additional file 2 Genes with ≤ -20 fold change between astrocyte and control retina Click here for file

Additional file 3 C57BL/6J astrocyte vs. DBA/2J astrocyte fold change Click here for file

Additional file 4 Oligonucleotides used for Real-Time qRT-PCR Click here for file

Additional file 5 Genes of principal component 1 (PC1) Click here for file

Additional file 6 Genes of principal component 2 (PC2) Click here for file

Additional file 7 Genes of principal component 3 through 5 (PC3-PC5) Click here for file

Additional file 8 **Network map of PC3 negative along with candidate genes**. This is a correlation network of 38 genes in PC3 negative with common bands as well as the genes that are candidates as modulator genes (yellow). Red lines represent ≥ 0.7 correlation; blue lines represent ≤ -0.7 correlation. Click here for file

Additional file 9 Candidate genes for PC3 negative QTL Click here for file
